# Yangqing Chenfei formula alleviates silica-induced pulmonary inflammation in rats by inhibiting macrophage M1 polarization

**DOI:** 10.1186/s13020-023-00787-9

**Published:** 2023-06-28

**Authors:** Xinrong Tian, Yu Wei, Runsu Hou, Xinguang Liu, Yange Tian, Peng Zhao, Jiansheng Li

**Affiliations:** 1 Henan Key Laboratory of Chinese Medicine for Respiratory Disease, Henan University of Chinese Medicine, Zhengzhou, 450046 Henan Province China; 2 Collaborative Innovation Center for Chinese Medicine and Respiratory Diseases Co-Constructed By Henan Province & Education Ministry of P.R. China, Zhengzhou, 450046 Henan Province China; 3 Academy of Chinese Medical Sciences, Henan University of Chinese Medicine, Zhengzhou, 450000 China; 4 Department of Respiratory Diseases, The First Affiliated Hospital of Henan University of Chinese Medicine, Zhengzhou, 450000 China

**Keywords:** Silicosis, Yangqing Chenfei formula, Macrophage polarization, Inflammation, Network pharmacology, Transcriptomic

## Abstract

**Background:**

Yangqing Chenfei formula (YCF) is a traditional Chinese medicine formula for early-stage silicosis. However, the therapeutic mechanism is unclear. The purpose of this study was to determine the mechanism for the effects of YCF on early-stage experimental silicosis.

**Methods:**

The anti-inflammatory and anti-fibrotic effects of YCF were determined in a silicosis rat model, which was established by intratracheal instillation of silica. The anti-inflammatory efficacy and molecular mechanisms of YCF were examined in a lipopolysaccharide (LPS)/interferon (IFN)-γ-induced macrophage inflammation model. Network pharmacology and transcriptomics were integrated to analyze the active components, corresponding targets, and anti-inflammatory mechanisms of YCF, and these mechanisms were validated in vitro.

**Results:**

Oral administration of YCF attenuated the pathological changes, reduced inflammatory cell infiltration, inhibited collagen deposition, decreased the levels of inflammatory factors, and reduced the number of M1 macrophages in the lung tissue of rats with silicosis. YCF5, the effective fraction of YCF, significantly attenuated the inflammatory factors induced by LPS and IFN-γ in M1 macrophages. Network pharmacology analysis showed that YCF contained 185 active components and 988 protein targets, which were mainly associated with inflammation-related signaling pathways. Transcriptomic analysis showed that YCF regulated 117 reversal genes mainly associated with the inflammatory response. Integrative analysis of network pharmacology and transcriptomics indicated that YCF suppressed M1 macrophage-mediated inflammation by regulating signaling networks, including the mTOR, mitogen-activated protein kinases (MAPK), PI3K-Akt, NF-κB, and JAK-STAT signaling pathways. In vitro studies confirmed that the active components of YCF significantly decreased the levels of p-mTORC1, p-P38, and p-P65 by suppressing the activation of related-pathways.

**Conclusion:**

YCF significantly attenuated the inflammatory response in rats with silicosis via the suppression of macrophage M1 polarization by inhibiting a “multicomponent-multitarget-multipathway” network.

## Background

Silicosis, which exhibits high morbidity and mortality rates in developing countries, is an irreversible occupational respiratory disease caused by long-term inhalation of crystalline silica dust [1, 2]. Silicosis is characterized by chronic inflammation and progressive interstitial fibrosis [3]. The pathogenesis of silicosis is unclear, thus, no effective clinical treatments to retard the progression of silicosis are available [4]. Pulmonary alveolar macrophages are the predominant cells involved in the development of silicosis [5]. When silica enters the lung, it can stimulate macrophages to engulf silica particles, resulting in M1 polarization and the induction of inflammation and tissue damage due to the release of large amounts of pro-inflammatory mediators, including interleukin-1β (IL-1β), IL-6, tumor necrosis factor-α (TNF-α), and cyclooxygenase-2 (COX-2) [6]. Macrophages cannot completely eliminate the phagocytosed silica dust due to the special structure of silica. The accumulation of silica in cells eventually leads to macrophages rupture, which further enhance inflammation and injury [7]. Prolonged inflammation and the resulting injury initiate the tissue repair. Activated macrophages can also release pro-fibrotic cytokines, such as transforming growth factor and vascular endothelial growth factor (VEGF), that initiate tissue repair and fibrosis [8–12]. Thus, Macrophage-orchestrated inflammation, injury and fibrosis play a key role in the pathological changes of silicosis. Membrane surface receptors, such as Toll-like receptors (TLRs), recognize silica and trigger inflammatory signaling cascade, which leads to the activation of various protein kinases and transcription factors, including mitogen-activated protein kinases (MAPK), nuclear factor-kappa B (NF-κB), and activator protein 1 [13–16], and the subsequent release of inflammatory cytokines, such as TNF-α, IL-1β, and IL-6. Thus, inhibiting the inflammatory signaling cascade can alleviate macrophage-mediated inflammation [17].

Yangqing Chenfei formula (YCF), a Chinese medicine for the clinical treatment of silicosis, is composed of 10 medicinal herbs. Previous study demonstrated that YCF exerts beneficial therapeutic effects against silicosis by alleviating clinical symptoms, such as wheezing, shortness of breath, and fatigue, and improving lung function, exercise tolerance, and the quality of life of patients [18]. However, due to the various components contained in YCF, the effective substances and their therapeutic mechanisms are difficult to identify. Target prediction, statistical analysis network-based techniques, and bioinformatics analysis were integrated with in vivo and vitro experimental validation and network pharmacology to analyze the systematic targets and mechanisms of multiple substances. Traditional Chinese medicine (TCM) network pharmacology is committed to exploring the mechanism of TCM based on the strategy of “multicomponent-multitarget-multipathway”. In addition, evaluating gene expression profiles with transcriptomics may yield further insight into molecular process regulated by TCM at the system level. Hence, integrating network pharmacology and transcriptomics may be an efficient approach to identifying the effective components and potential therapeutic mechanisms of YCF in silicosis. We investigated the therapeutic and anti-inflammation effects of YCF in vivo and in vitro, and integrated network pharmacology and transcriptomics to reveal the active substances and anti-inflammation effects of YCF on macrophages activation.

## Methods

### Animals and reagents

Thirty-two male Sprague Dawley rats, weighing between 180 and 220 g, were purchased from the Experimental Animal Center of Vital River in Beijing, China. The animals were housed in conventional laboratory conditions, at a temperature of 25 °C and a humidity of 55%, with unrestricted access to food and water.

Silicon dioxide was purchased from Sigma-Aldrich, (Cat. S5631, Darmstadt, Germany). Yangqing Chenfei formula (Patent No. CN112057580A) was provided by the Department of Pharmacology at Henan University of Chinese Medicine’s First Affiliated Hospital in China. The botanical drug compositions of YCF are shown in Table 1. Tetrandrine tablets (SFDA approval No.H33022075, Jinaikang, China, 20 mg per tablet) were purchased from CONBA Bio-Pharm (Zhejiang, China). The following ELISA kits were used: Rat IL-1β High Sensitivity ELISA Kit (Cat. EK301BHS, Multi Sciences, Hangzhou, China), Rat IL-6 ELISA Set (Cat. 550319, BD, San Diego, United States), and ELISA MAX™ Deluxe Set Rat TNF-α (Cat. 438204, BioLegend, San Diego, United States).

**Table 1:** The main herbal drugs contained in Yangqing Chenfei formula

Plant names	Genus	Family	Authorities	
*Ophiopogon japonicus* (Thunb.) Ker Gawl	*Ophiopogon*	*Asparagaceae*	Ophiopogonis Radix	
*Panax quinquefolius* L	*Panax*	*Araliaceae*	Panacis Quinquefolii Radix	
*Scrophularia ningpoensis* Hemsl	*Scrophularia*	*Scrophulariaceae*	Scrophulariae Radix	
*Fritillaria thunbergii* Miq	*Fritillaria*	*Liliaceae*	Fritillariae Thunbergii Bulbus	
*Curcuma aromatica* Salisb	*Curcuma*	*Zingiberaceae*	Curcumae Radix	
*Platycodon grandiflorus* (Jacq.) A.DC	*Platycodon*	*Campanulaceae*	Platycodonis Radix	

The following antibodies were used: phospho-mTOR (Ser2448) antibody (Cat. GTX132803, GeneTex, United States), mTOR antibody (Cat. GTX101557, GeneTex, United States), phospho-p38 MAPK antibody (Cat. 4511S, Cell Signaling Technology, Boston, United States), p38 MAPK antibody (Cat. 8690S, Cell Signaling Technology, Boston, United States), phospho-NF-κB p65 (Cat. 3033S, Cell Signaling Technology, Boston, United States), NF-κB p65 antibody (Cat. 8242S, Cell Signaling Technology, Boston, United States), HRP-conjugated Affinipure Goat Anti-Rabbit lgG (Cat. SA00001-2, Proteintech, Chicago, United States), and CD68 antibody (Cat. DF7518, Affinity, United States), iNOS antibody (Cat. No. AF0199, Affinity, United States).

### Preparation of YCF extract

To obtain YCF extract, Ophiopogonis Radix, Scrophulariae Radix, Platycodonis Radix and so on were decocted and filtered twice with pure water. Panacis Quinquefolii Radix, Fritillariae Thunbergii Bulbus, Curcumae Radix, and so on were extracted and filtered twice with 60% ethanol. The alcohol extract was mixed with the aforementioned solution. All components were crushed to obtain a fine powder after the extracted solution was finely powdered and dried under decreased pressure.

### Silicosis rat model, and drug administration

Eight rats per group were randomly assigned to the Normal group, Model group, YCF treatment group, and TET treatment group. Rats with silicosis were anesthetized with sodium pentobarbital, and 50 mg of silica suspension were intratracheally instilled, while an equal volume of normal saline was instilled in the Normal group [19]. The YCF treatment group received intragastric YCF (3.3663 g/kg per day; 0.84 mL/100 g per day, dissolved in normal saline), and the TET treatment group received intragastric TET (27 mg/kg per day, dissolved with normal saline) for 14 days. Rats in the Normal and Model groups respectively intragastrical treated with 2 mL normal saline. All rats were slaughtered on day 15. The experimental scheme was permitted by the First Affiliated Hospital of Henan University of Chinese Medicine's Experimental Animal Ethics Committee (Zhengzhou, China).

### Cell culture and treatment

The murine alveolar macrophage cell line (MH-S) was acquired from the Chinese Academy of Sciences in Shanghai, China, and was maintained in RPMI 1640 (Cat. 31,800, Solarbio, Beijing, China) media with 10% fetal bovine serum (Cat. S711-011S, Lonsera, South America) at 37 °C, in a 5% CO_2_ incubator. MH-S cells were seeded in 6-well culture plates at a concentration of 1 × 10 ^6^ cells/well for 1 day. After pretreatment with various concentrations of YCF (50 μg/mL, 25 μg/mL) and the active compounds of YCF5 for 3–6 h, and cells were treated with 100 ng/mL lipopolysaccharide (LPS) (Cat. L2880, Sigma-Aldrich, Darmstadt, Germany) and 2 ng/mL interferon (IFN)-γ (Cat. 315-05-100UG, Peprotech, United States) for 12 h.

### Histopathological analysis

Lung tissue samples were preserved in a 4% paraformaldehyde solution and then embedded in paraffin. Hematoxylin–eosin (HE) and Masson’s Trichrome stains were applied to 4-µm-thick slices of tissue paraffin blocks, and the stained sections were observed under a light microscope. Lung cellular infiltration and collagen fiber levels were analyzed in a double-blind manner by three experienced investigators based on the histopathological evaluation criteria for silicosis (Szapiel score and Ashcroft score) [20, 21].

### Immunohistochemistry analysis

Lung tissue sections were deparaffinized, rehydrated, and blocked with 5% BSA for 0.5 h at 37 ℃, after washing 3 times with phosphate-buffered saline (PBS), and slide sections were incubated overnight at 4 ℃ with anti-iNOS, anti-CD68 antibodies. After washing with PBS, the slides were incubated at 37 °C with the secondary antibody, and followed by staining with DAB chromogenic solution. IPP 6.0 was used to detect integral optical density.

### Drug isolation

Firstly, 10 g YCF was dissolved with methanol and pure water. Secondly, 100 g DIAION HP-20 adsorbent resin (Mitsubishi, Japan) was submerged in absolute alcohol overnight on the chromatographic column, and followed by washing with pure water until no alcohol remained. Thirdly, The YCF solution was fully mixed with the HP-20 resin, and the chromatographic column was eluted with water, and 10%, 30%, 60%, and 95% industrial alcohol. Finally, the eluents were named YCF1, YCF2, YCF3, YCF4, and YCF5, respectively, and were distilled and dissolved in DMSO at a concentration of 100 mg/ml.

### Quantitative real-time PCR

Total RNA was extracted from MH-S cell samples using QIAzol reagent (Cat. 79,306, QIAGEN, United States). HiScript II Q RT SuperMix (Cat. R223, Vazyme, Nanjing, China) was used to reverse transcribe equal concentrations of mRNAs into cDNAs for qPCR. PCR was performed using SYBR (Cat. Q711, Vazyme, Nanjing, China) and the QuantStudio 6 RT-PCR detection system. Total RNA extraction, cDNA synthesis, and RT-PCR analysis were all performed according to the manufacturers’ instructions. Relative expression levels of IL-1β, IL-6, TNF-α, and COX-2 were calculated by normalizing to GAPDH. The results were analyzed using the 2 − ΔΔCt method. The specific primer sequences are listed in Table 2.

**Table 2:** The primers in RT-PCR

Gene	Forward	Reverse
IL-1β	GAAATGCCACCTTTTGACAGTG	TGGATGCTCTCATCAGGACAG
IL-6	CTGCAAGAGACTTCCATCCAG	AGTGGTATAGACAGGTCTGTTGG
TNF-α	CTGAACTTCGGGGTGATCGG	GGCTTGTCACTCGAATTTTGAGA
COX-2	TTCCAATCCATGTCAAAACCGT	AGTCCGGGTACAGTCACACTT
GAPDH	AGGTCGGTGTGAACGGATTTG	GGGGTCGTTGATGGCAACA

### Western blotting

Proteins were extracted from samples using 200 μl RIPA, and adjusted to equal concentrations utilizing a BCA Kit. Denatured protein samples were separated by gel electrophoresis and transferred to PVDF membranes. Following overnight incubation at 4 °C with corresponding primary antibodies, the membranes were incubated for 1 h at room temperature with HRP-conjugated anti-rabbit secondary antibodies. Bands were observed and quantified using Image Lab software. The relative protein level was normalized by GAPDH.

### Identification of compounds in YCF5

#### Chromatography conditions

To identify the YCF5 compounds in extracts, YCF5 samples were subjected to liquid chromatography (LC)-mass spectrometry (MS) using a Dionex Ultimate 3000 UPLC system coupled to a Thermo Scientific Q Exactive Orbitrap mass spectrometer. Samples were loaded onto the Phenomenex Synergi Polar-RP (2 × 150 mm, 4 μm) at 40 ℃. The flow rate was 0.3 mL/min. Mobile phase A was composed of water and 0.1% formic acid, and mobile phase B was composed of acetonitrile and 0.1% formic acid. The optimum gradient elution conditions were as follows: 0% B (0–5 min), linear gradient from 0% B to 5% B (5−7 min), 5% B to 20% B (7−10 min), 20% B to 25% B (10−20 min), 25% B to 50% B (20−23 min), and 50% B to 100% B (23−40 min), 100% B for a further 3 min (40−43 min), then back to 0% B (43−45 min), 0% B for 5 min, and stop at 50 min. The injection volume was 5.00 μL.

#### MS conditions

MS necessitates the use of heated electrospray ionization. The spray voltage was adjusted at 3500 V for the positive ion mode and 2800 V for the negative ion mode. Sheath gas was set to 40 Arb, while Aux gas was set to 10 Arb. The capillary temperature was set to 325 °C, while the Aux gas heater temperature was set at 300 °C. In the Orbitrap, full scans from m/z 100 to 1500 were performed at a resolution of 70 K for quantification, an automatic gain control (AGC) target of 3106, and a maximum injection time of 200 ms. Fragment identification and quantification were performed using the parallel reaction monitoring (PRM) mode. The resolution of Target MS2 scans in PRM was 17.5 K, the isolation breadth was 4.0 Da, the AGC target was 2 × 10^5^, and the maximum injection duration was 100 ms. The 185 identified YCF5 compounds are listed in Table 3.

**Table 3:** The identified compounds of YCF5

		Compound	RT	Formula
1	pos	ArginineR	1.28	C6H14N4O2
2	pos	InositolR	1.30	C6H12O6
3	pos	D-Fructose or its isomer	1.33	C6H12O6
4	neg	Glutamic acid	1.33	C5H9NO4
5	neg	Mannitol	1.35	C6H14O6
6	pos	Panose	1.36	C18H32O16
7	pos	γ-Lactone galactoate or its isomer	1.36	C6H10O6
8	pos	5-Hydroxymethylfurfural or its isomer	1.38	C6H6O3
9	pos	Xylitol	1.38	C5H12O5
10	neg	D-Fructose or its isomer	1.41	C6H12O6
11	neg	Quinic acid	1.48	C7H12O6
12	neg	γ-Lactone galactoate or its isomer	1.58	C6H10O6
13	neg	L-Malic acidR	1.64	C4H6O5
14	pos	SynephrineR	1.78	C9H13NO2
15	neg	Gallic acidR	4.17	C7H6O5
16	neg	Protocatechuic acid isomer	11.66	C7H6O4
17	neg	Zedoarolide B	12.58	C15H22O5
18	neg	OxypaeoniflorinR	13.97	C23H28O12
19	neg	Chlorogenic acid/Cryptochlorogenic acid/5-Caffeoylquinic acid/3-Caffeoylquinic acid/4-Caffeoylquinic acid	14.03	C16H18O9
20	neg		14.74	C9H8O4
21	neg	EsculetinR	15.01	C9H6O4
22	neg	Diosmetin-6,8-di-C-glucoside	15.33	C28H32O16
23	pos	Lactiflorin or its isomer	15.82	C23H26O10
24	neg	Mudanpioside E	15.83	C24H30O13
25	neg	PaeoniflorinR	15.83	C23H28O11
26	neg	3-O-trans-coumaroylquinic acid	15.85	C16H18O8
27	neg	3,4-Dicaffeoylquinic acid/3,5-Dicaffeoylquinic acid/4,5-Dicaffeoylquinic acid/Cynarin	16.04	C25H24O12
28	neg	RutinR	16.88	C27H30O16
29	pos	Verticinone-3-glucoside or its isomer	16.89	C33H53NO8
30	neg	EriocitrinR	16.98	C27H32O15
31	pos	Astin G	17.18	C25H35N5O6
32	neg	ActeosideR	17.31	C29H36O15
33	neg	HyperosideR	17.58	C21H20O12
34	neg	1,2,4,6-Tetra-O-Galloyl-Î’-D-Glucose or its isomer	17.64	C34H28O22
35	pos	Trihydroxy-dimethoxyflavone	17.81	C17H14O7
36	neg	Jaceosidin-7-O-glucoside	17.96	C23H24O12
37	neg	cis-Acteoside/Isoacteoside	17.97	C29H36O15
38	neg	1,2,4,6-Tetra-O-Galloyl-Î’-D-Glucose or its isomer	18.07	C34H28O22
39	pos	Verticinone-3-glucoside or its isomer	18.34	C33H53NO8
40	pos	Prunin or its isomer	18.42	C21H22O10
41	pos	NarirutinR	18.53	C27H32O14
42	pos	Hesperetin or its isomer	18.60	C16H14O6
43	neg	6″-O-(p-coumaroyl)harpagide/8-O-(p-courmaroyl)harpagide	18.63	C24H30O12
44	pos	3β-Hydroxylup-20(29)-en-30-al	18.72	C30H48O2
45	pos	Rhoifolin	18.76	C27H30O14
46	neg	Neodiosmin	18.93	C28H32O15
47	neg	3,4-Dicaffeoylquinic acid/3,5-Dicaffeoylquinic acid/4,5-Dicaffeoylquinic acid/Cynarin	19.11	C25H24O12
48	pos	Peimisine or its isomer	19.43	C27H41NO3
49	pos	Peimisine or its isomer	19.71	C27H41NO3
50	neg	Prunin or its isomer	19.73	C21H22O10
51	pos	Meranzin/Isomeramazin	19.74	C15H16O4
52	neg	3,4-Dicaffeoylquinic acid/3,5-Dicaffeoylquinic acid/4,5-Dicaffeoylquinic acid/1,5-Dicaffeoylquinic acid	20.00	C25H24O12
53	neg	Scrophuside	20.06	C30H38O15
54	pos	Homoplantaginin/Chrysoflavin -7-O-glucoside or its isomer	20.23	C22H22O11
55	neg	Lactiflorin or its isomer	20.30	C23H26O10
56	pos	Ophiopogonanone B	20.41	C18H18O5
57	pos	PeimineR	20.61	C27H45NO3
58	neg	Hesperetin or its isomer	20.68	C16H14O6
59	pos	20(S)-Ginsenoside Rg3/Ginsenoside F2/20(S)-Ginsenoside Rg3 or their isomer	20.75	C42H72O13
60	pos	Peimisine or its isomer	20.89	C27H41NO3
61	pos	Verticinone-N-Oxide	20.93	C27H43NO4
62	neg	Sudachiin C/Sudachiin B or their isomer	21.17	C30H34O17
63	neg	Cistanoside D or its isomer	21.60	C31H40O15
64	neg	BaicalinR	21.72	C21H18O11
65	pos	Paeonol isomer	21.89	C9H10O3
66	neg	HarpagosideR	21.92	C24H30O11
67	neg	Deapioplatycodin D	21.94	C52H84O24
68	neg	Benzoyl-Oxypaeoniflorin/Mudanpioside C	22.00	C30H32O13
69	pos	PeiminineR	22.09	C27H43NO3
70	neg	Platycodin D	22.31	C57H92O28
71	neg	Platycoside B/Platycoside C	22.46	C54H86O25
72	neg	Cistanoside D or its isomer	22.71	C31H40O15
73	pos	Platyconic acid C or its isomer	22.72	C52H82O25
74	neg	Polygalacin D	22.73	C57H92O27
75	neg	Benzoyl-Oxypaeoniflorin/Mudanpioside C	22.79	C30H32O13
76	pos	Platycoside K/Platycoside L	22.87	C42H68O17
77	pos	Pseudoginsenoside RT2	23.01	C41H70O14
78	pos	20(R)-Ginsenoside Rg2	23.03	C42H72O13
79	pos	20(R)-Ginsenoside Rh1	23.03	C36H62O9
80	pos	Cucurbitacin D or its isomer	23.09	C30H44O7
81	pos	Platyconic acid C or its isomer	23.09	C52H82O25
82	neg	Platyconic acid A	23.11	C57H90O29
83	pos	Platycogenic Acid A/Platycogenic Acid B	23.12	C30H46O8
84	pos	Poncirin/Didymin	23.20	C28H34O14
85	neg	Platycoside B/Platycoside C	23.47	C54H86O25
86	pos	Isoverticine/Zhebeinine	23.52	C27H45NO3
87	pos	Pseudoginsenoside RT5	23.55	C36H62O10
88	pos	Pseudoginsenoside F11	23.61	C42H72O14
89	neg	Astersaponin A/Astersaponin E	23.62	C67H108O34
90	pos	Verticinone-3-glucoside or its isomer	23.86	C33H55NO7
91	pos	N-Trans-Feruloyltyramine or its isomer	23.92	C18H19NO4
92	neg	Sudachiin C/Sudachiin B or their isomer	23.93	C30H34O17
93	pos	Platycosaponin A	24.47	C42H68O16
94	pos	Panaquinquecol 1 or its isomer	24.71	C18H28O3
95	pos	Suchengbeisine	24.90	C27H43NO3
96	pos	Furanogermenone/Glechomanolide/Turmeronol B/ Turmeronol A	24.92	C15H20O2
97	pos	Curcumenol/Isocurcumenol/Neocurcumenol	25.29	C15H22O2
98	pos	Platycoside K/Platycoside L	25.59	C42H68O17
99	pos	Melitidin	26.01	C33H40O18
100	neg	3-O-glucopyranosyl platycodigenin	26.23	C36H58O12
101	pos	Platycodigenin	26.26	C30H48O7
102	pos	20(S)-Ginsenoside Rg3/Ginsenoside F2/20(S)-Ginsenoside Rg3 or their isomer	26.30	C42H72O13
103	pos	Ginsenoside Rd or its isomer	26.30	C48H82O18
104	pos	Ginsenoside Rg5/Ginsenoside Rk1 or their isomer	26.30	C42H70O12
105	pos	Taraxerone or its isomer	26.30	C30H48O
106	pos	Ginsenoside Rb1R	26.34	C54H92O23
107	pos	Dibutyl sebacate or its isomer	26.92	C18H34O4
108	pos	Platycosaponin A	27.21	C42H68O16
109	pos	20(S)-Ginsenoside Rg3/Ginsenoside F2/20(S)-Ginsenoside Rg3 or their isomer	27.46	C42H72O13
110	pos	Cucurbitacin D or its isomer	27.60	C30H44O7
111	pos	Benzoylpaeoniflorin	27.68	C30H32O12
112	pos	Fritillarizine/Puqiedinone/Eduardine/Zhebeirine	27.85	C27H43NO2
113	pos	Ginsenoside Rh4/Ginsenoside Rk3	27.89	C36H60O8
114	pos	Ginsenoside Rk2/Ginsenoside Rh3 or their isomer	28.01	C36H60O7
115	pos	PaeonolR	28.07	C9H10O3
116	pos	Cucurbitacin D or its isomer	28.68	C30H44O7
117	neg	Yunganoside A1/Yunganoside B1/Yunganoside C1	28.99	C48H76O19
118	pos	Ebeiedine/Puqiedine/N-demethylpuqietinone/Eduardinine	29.08	C27H45NO2
119	pos	5-Hydroxy-7,8,4′-Trimethoxyflavanone or its isomer	29.10	C18H16O6
120	pos	5-Hydroxy-6,7,8,3′,4′-Pentamethoxyflavone	29.26	C20H20O8
121	pos	Monohydroxy-pentamethoxyflavone	29.42	C20H20O8
122	neg	Naringenin	29.47	C15H12O5
123	pos	Furanogermenone/glechomanolide/turmeronol B/ turmeronol A	29.80	C15H20O2
124	neg	Diosmetin	29.98	C16H12O6
125	pos	Taraxerone or its isomer	30.29	C30H48O
126	pos	Ginsenoside Rg5/Ginsenoside Rk1 or their isomer	30.41	C42H70O12
127	pos	Monohydroxy-tetramethoxyflavone	30.49	C19H18O7
128	pos	Monohydroxy-pentamethoxyflavone	30.74	C20H20O8
129	neg	Hesperetin or its isomer	30.80	C16H14O6
130	pos	Curcumenol/Isocurcumenol/Neocurcumenol	30.93	C15H22O2
131	pos	beta-Turmerone	30.97	C15H22O
132	pos	3-O-(-d-arabinopyranosyl-(1 → 6)-d-glucopyranosyl)-2,3,16-trihydroxyolean-12-en-28-oic acid	31.42	C41H66O14
133	pos	Ginsenoside Rd or its isomer	32.03	C48H82O18
134	neg	Chikusetsusaponin IVa/Zingibroside R1/Saponin Rb-4	32.06	C42H66O14
135	pos	Monohydroxy-pentamethoxyflavone	32.57	C20H20O8
136	pos	Sinensetin or their isomer	34.01	C20H20O7
137	pos	Monohydroxy-hexamethoxyflavone	34.09	C21H22O9
138	pos	20(S)-Ginsenoside Rg3/Ginsenoside F2/20(S)-Ginsenoside Rg3 or their isomer	34.96	C42H72O13
139	pos	Ophiopogonanone F or its isomer	35.00	C20H22O7
140	pos	6-Gingerol	36.76	C17H26O4
141	pos	Sinensetin or their isomer	36.85	C20H20O7
142	pos	Ophiopogonanone F or its isomer	37.77	C20H22O7
143	pos	Tetramethyl-O-scutellarein/Tetramethyl-O-isoscutellarein	38.17	C19H18O6
144	pos	Panaquinquecol 1 or its isomer	39.44	C18H28O3
145	pos	NobiletinR	39.61	C21H22O8
146	pos	Panaquinquecol 1 or its isomer	40.20	C18H28O3
147	pos	Limonin	40.66	C26H30O8
148	pos	Ginsenoside Rg5/Ginsenoside Rk1 or their isomer	40.95	C42H70O12
149	pos	Taraxerone or its isomer	40.95	C30H48O
150	pos	Ginsenoside Rk2/Ginsenoside Rh3 or their isomer	41.03	C36H60O7
151	neg	Chikusetsusaponin IVa/Zingibroside R1/Saponin Rb-4	41.06	C42H66O14
152	pos	20(S)-Ginsenoside Rg3/Ginsenoside F2/20(S)-Ginsenoside Rg3 or their isomer	41.09	C42H72O13
153	pos	Tetramethyl-O-scutellarein/Tetramethyl-O-isoscutellarein	41.09	C19H18O6
154	pos	Paeonol isomer	41.37	C9H10O3
155	pos	5,7,4′-Trimethoxyflavone	41.58	C18H16O5
156	pos	Ophiopogonanone E	42.95	C19H20O7
157	pos	TangeretinR	43.03	C20H20O7
158	pos	Ginsenoside Rs4/Ginsenoside Rs5	43.93	C44H72O13
159	pos	Nomilin	43.99	C28H34O9
160	neg	Calenduloside E	44.93	C36H56O9
161	pos	Ginsenoside Rk2/Ginsenoside Rh3 or their isomer	45.22	C36H60O7
162	pos	Taraxerone or its isomer	45.22	C30H48O
163	pos	Ginsenoside Rg5/Ginsenoside Rk1 or their isomer	45.24	C42H70O12
164	pos	Ginsenoside Rg5/Ginsenoside Rk1 or their isomer	45.57	C42H70O12
165	pos	Monohydroxy-pentamethoxyflavone	45.57	C20H20O8
166	pos	2-Monolinolein	45.59	C21H38O4
167	pos	Taraxerone or its isomer	45.59	C30H48O
168	pos	Ginsenoside Rk2/Ginsenoside Rh3 or their isomer	45.63	C36H60O7
169	neg	3-O-glucopyranosyl polygalacic acid	45.95	C36H58O11
170	pos	Panaquinquecol 1 or its isomer	46.55	C18H28O3
171	pos	Monohydroxy-hexamethoxyflavone	46.80	C21H22O9
172	pos	Ginsenoside Rs4/Ginsenoside Rs5	47.42	C44H72O13
173	pos	Ophiopogonanone F or its isomer	47.76	C20H22O7
174	pos	Ginsenoside Rk2/Ginsenoside Rh3 or their isomer	48.86	C36H60O7
175	pos	Stearidonic acid or its isomer	48.86	C18H28O2
176	pos	Stearidonic acid or its isomer	49.07	C18H28O2
177	pos	Stearidonic acid or its isomer	49.35	C18H28O2
178	pos	Dibutyl phthalate/Isobutyl phthalate or their isomer	49.76	C16H22O4
179	pos	Dibutyl phthalate/Isobutyl phthalate or their isomer	50.09	C16H22O4
180	pos	Panaquinquecol 1 or its isomer	51.70	C18H28O3
181	pos	Erucic amide or its isomer	54.63	C22H43NO
182	pos	Soyacerebroside I	56.49	C40H75NO9
183	pos	Erucic amide or its isomer	56.55	C22H43NO
184	pos	Bis (2-ethylhexyl) phthalate or its isomer	57.40	C24H38O4
185	pos	Stigmasterol	62.30	C29H48O

#### Identification of YCF5-associated molecular targets

To predict the potential targets of YCF5, known ingredients in the above-mentioned identified compounds from MS were screened out of the PubChem database (https://pubchem.ncbi.nlm.nih.gov/) and DrugBank Online (https://go.drugbank.com/). These ingredients were considered potentially active compounds of YCF. Canonical SMILES for each compound were collected and entered into the Swiss Target Prediction database (http://www.swisstargetprediction.ch/) and STITCH database (http://stitch.embl.de) for target prediction, and the targets with a probability greater than 0 were saved. Finally, Cytoscape 3.9.0 software was used to construct and analyze the compound-target network.

### Transcriptomics analysis

#### cDNA synthesis

Total RNA was isolated using TRIzol^®^ Reagent (Cat. 15,596,026, Ambion, Carlsbad, United States). Paired-end libraries were created by following the RNA sample preparation guidance from the TruSeq™ RNA Sample Preparation Kit (Illumina, USA). In brief, poly-A mRNA molecules were isolated and fragmented. Using reverse transcriptase and random primers, the cleaved RNA fragments were transcribed into first strand cDNA. Second strand cDNA synthesis was performed using DNA Polymerase I and RNase H. The cDNA fragments were end-repaired, by adding a single “A” base, and enriched with PCR to generate the final cDNA library. To confirm the insert size and determine the concentration, purified libraries were measured using a Qubit® 2.0 Fluorometer (Life Technologies, USA) and verified using an Agilent 2100 Bioanalyzer (Agilent Technologies, USA). After diluting the library to 10 pM, cBot-constructed clusters were sequenced on the Illumina NovaSeq6000 (Illumina, USA).

#### Differential expression analysis

The R package edgeR was used to perform mRNA differential expression analysis. Differentially expressed RNAs with |log2fold change| values more than one and P values less than 0.05 were kept for further analysis. The decision to increase the sensitivity of this analysis in order to run a vast screening and discover candidate genes to be verified with a larger sample population is what inspired this approach. The identify candidate genes were classified as up-regulated or down-regulated according to the log2fold change greater than 0 or less than 0. The intersections between the up-regulated genes in the Model vs. Control group and the down-regulated genes in the YCF vs. Model group, and the down-regulated genes in the Model vs. Control group and the up-regulated genes in the YCF vs. Model group. These differential genes were identified as reversing genes.

#### Kyoto Encyclopedia of Genes and Genomes (KEGG) pathways enrichment and Gene Ontology (GO) enrichment analysis

ClueGO is a Cytoscape plug-in that enables the construction of a functionally grouped network from a large number of genes. Using the KEGG database, we performed pathway enrichment analysis of reversal genes to verify functional classes of reversal genes with statistical significance (p-value 0.05).The GO database was used to visualize the underlying biological calcium process, cellular component and molecular function of these reversal genes.

#### Protein–protein interaction (PPI) network construction

After removing duplicate values, we obtained 699 targets for the components contained in YCF and 117 reverse genes. To further explore the link between the target and reversal genes, we constructed a PPI network through STRING. Based on the putative targets and the reversal gene-associated signaling pathways, a Sankey diagram of the compound–target–enrichment pathway–reversal gene was established.

### Experimental docking of molecules

#### Processing and design of small molecules

The Minimum RMS Gradient was set to 0.01, the supplied small molecules were imported into ChemBio3D Ultra 21.0.0.28 for energy minimization, and the small molecules were saved in “pdb” format. The optimized small molecules were saved in “pdbqt” format and imported into AutodockTools-1.5.6 for hydrogenation, charge computation, charge distribution, and configuration of rotatable keys.

#### Protein preparation and processing

To eliminate the protein water of crystallization, the original ligand, and other impurities, the given protein was imported into Pymol 2.3.0. The protein structures were then imported into AutoDocktools (v1.5.6) for hydrogenation, charge calculation, charge distribution, and atomic type specification, and saved in the "pdbqt" format.

#### Making the parameter files

The parameters for each receptor were set using AutoDock Vina1.1.2 for docking (with a spacing of 0.375 between each grid point, Table 3), exhibitiveness: 8, and the other parameters were left at their default settings.

#### Results analysis

Interaction modes were analyzed using PyMOL23.0 and Discovery Studio based on the molecular docking results.

### Statistical analysis

All data are presented as means ± standard deviations. SPSS 26.0 software was used for statistical analyses. For multiple group comparisons, the results were analyzed using one-way ANOVA. Data were considered statistically significant when the P-value was less than 0.05.

## Results

### YCF significantly attenuates inflammation and fibrosis in the lungs of rats induced by silica

**Fig. 1:**
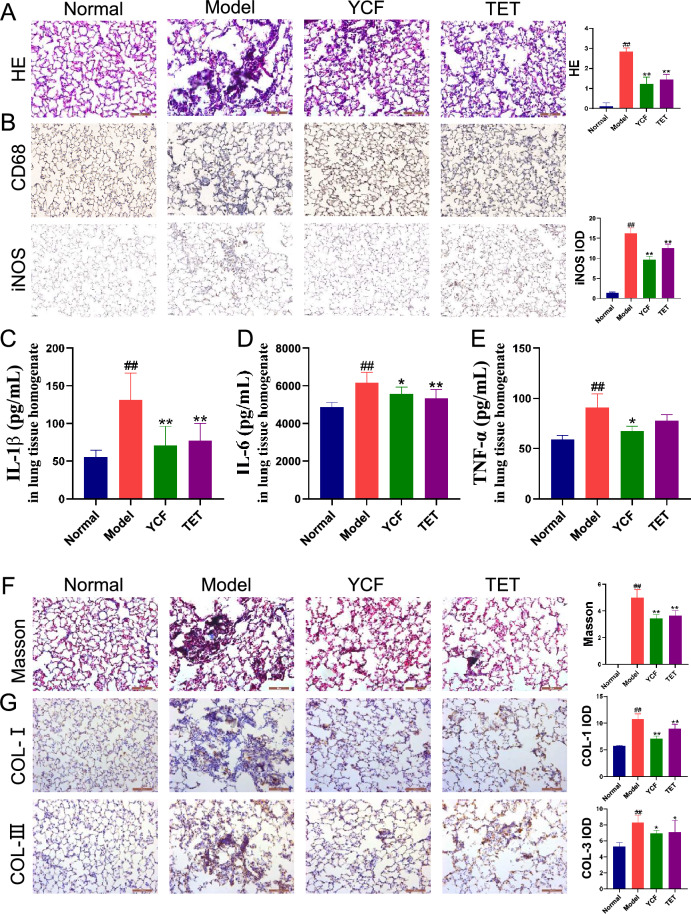
Yangqing Chenfei Formula (YCF) attenuates inflammatory cell infiltration, pathological damage, and collagen deposition in the lungs of rats with silicosis by decreasing the number of macrophages. **A** HE staining of lung tissue from rats with silicosis (200×). **B** Expression of CD68 and iNOS (200×) and the integral optical density of iNOS using immunohistochemistry. **C**–**E** The levels of IL-1β, IL-6, and TNF-α, respectively, in lung tissue homogenates. **F** Masson staining of lung tissue from rats with silicosis (200×) and collagen volume fraction. **G** Expression of COL-1 and COL-3 (200×) and the IOD of COL-1 and COL-3 using immunohistochemistry. All data are presented as mean ± SD (*n* = 6). ^#^*p* < 0.05, ^##^*p* < 0.01 *vs*. Normal rats; ^*^p < 0.05, ^**^p < 0.01 *vs*. Model rats

As shown in Fig. 1A, YCF treatment attenuated cell infiltration, and pathological changes and significantly reduced the HE score. IHC staining showed that CD68 and iNOS, which are pro-inflammatory markers in macrophages, were remarkably expressed in the lung of rats with silicosis (Fig. 1B). Treatment with YCF or TET significantly decreased these pro-inflammatory markers, and the effects of YCF were better than the effects of TET. Protein levels of IL-1β, IL-6, and TNF-α increased in the lungs of rats with silicosis and significantly decreased in YCF- and TET-treated rats (Fig. 1C–E). Furthermore, collagen deposition increased in the lungs of rats with silicosis, and YCF and TET treatment attenuated the increased collagen deposition (Fig. 1F). YCF and TET treatment obviously reduced type I and III collagen deposition in the lungs (Fig. 1G). Taken together, these data suggest that YCF treatment reduces the inflammatory response, lung injury, and subsequent fibrosis induced in the silicosis rat model.

### YCF suppresses inflammatory response in M1 macrophage

**Fig. 2:**
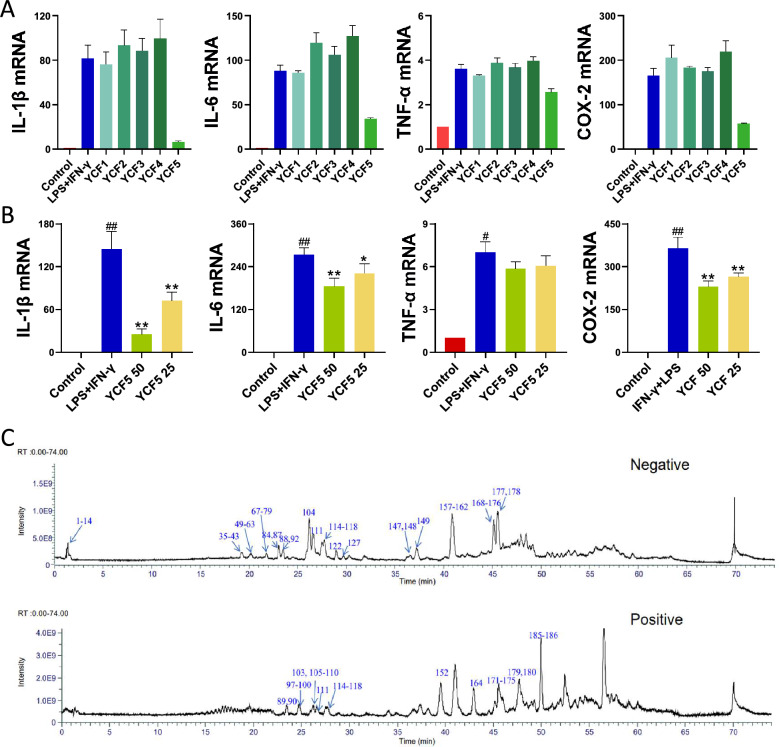
YCF5, the effective fragment of YCF, significantly inhibited M1 macrophage-induced inflammation by suppressing the release of pro-inflammatory factors. **A** mRNA expression of IL-1β, IL-6, TNF-α, and COX-2 in the different fractions of YCF. **B** Expression of IL-1β, IL-6, TNF-α, and COX-2 in different concentrations of YCF5. **C** The total ion chromatogram of YCF5 in negative and positive mode. All data are presented as the mean ± SD (*n* = 3). ^#^*p* < 0.05, ^##^*p* < 0.01 *vs*. Control group; ^*^p < 0.05, ^**^p < 0.01 *vs*. Model group

M1 Macrophage-mediated inflammation is the initial cause of silicosis progression. YCF treatment reduced macrophage infiltration and activation in the lung of rats with silicosis. Thus, we investigated the effects of YCF on the M1 macrophage polarization, the pro-inflammation phenotype, in vitro. As shown in Fig. 2A and B, LPS and IFN-γ stimulated the expression of pro-inflammatory factors in macrophages, including IL-1β, IL-6, TNF-α, COX-2, and YCF5, the active substance of YCF, significantly suppressed the expression of these inflammatory factors in a concentration-dependent manner. Using LC–MS/MS in negative and positive modes, to identify the active compounds of YCF5, and obtained a total of 185 active compounds by ion chromatogram, which may act as anti-inflammatory components of YCF5 (Fig. 2C and Table 3). These results demonstrated that YCF5, the active fraction of YCF, suppresses inflammatory response in M1 macrophage.

### Network pharmacology analysis of YCF5

**Fig. 3:**
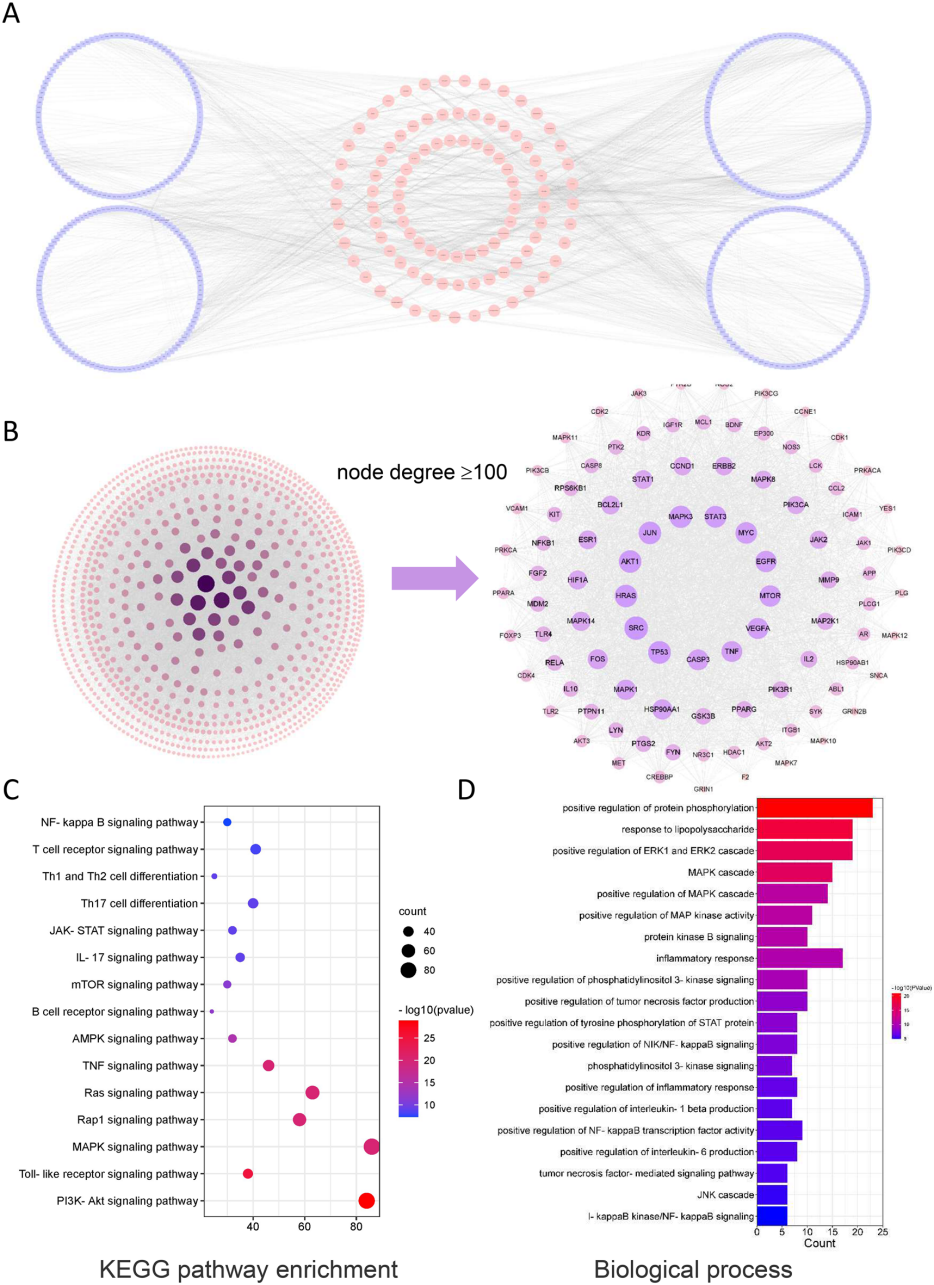
Target analysis of active components. **A** Compound-target network of YCF. The circular nodes are the YCF5 compounds, and the square nodes are the targets of the YCF5 compounds. **B** Protein–protein interaction (PPI) network of the targets of YCF5 active components. **C** Kyoto Encyclopedia of Genes and Genomes (KEGG) analysis of the targets of YCF5. **D** Biological process analysis of gene ontology for the targets of YCF5

TCM formulas are characterized by multiple components, multiple targets and multiple pathways. Hence, we applied network pharmacology to investigate the active compounds, targets and pathways of YCF5. Using the Swiss Target Prediction and STITCH databases, 988 targets were identified for the 185 compounds contained in YCF5. A compound-target network containing the 185 active ingredients and 988 targets was constructed (Fig. 3A). The PPI network of these targets consisted of 930 nodes and 20,103 edges. To further explore the potential relationship of these genes, we used the CytoNCA plug-in of Cytoscape to find the critical nodes, including Akt1, Trp53, Tnf, Mapk3, Src, Jun, Hras, Egfr, Stat3, Mapk14 (Fig. 3B). KEGG enrichment and GO function analyses of the genes with node degree values greater than 100 were performed using the CluGO plug-in of Cytoscape. As shown in the Fig. 3C and D, a total of 165 KEGG pathways and 800 biological processes, which were mostly related to immune response and inflammation were identified. The pathways included the T cell receptor signaling pathway, Th1 and Th2 cell differentiation, Toll-like receptor signaling pathway, and the positive regulation of interleukin-1β and interleukin-6 production, and the positive regulation of inflammatory response. These results indicated that the biological function of YCF5-related targets was mainly associated with immune response and inflammation.

### Transcriptomics analysis results of YCF5-treated M1 macrophages

**Fig. 4:**
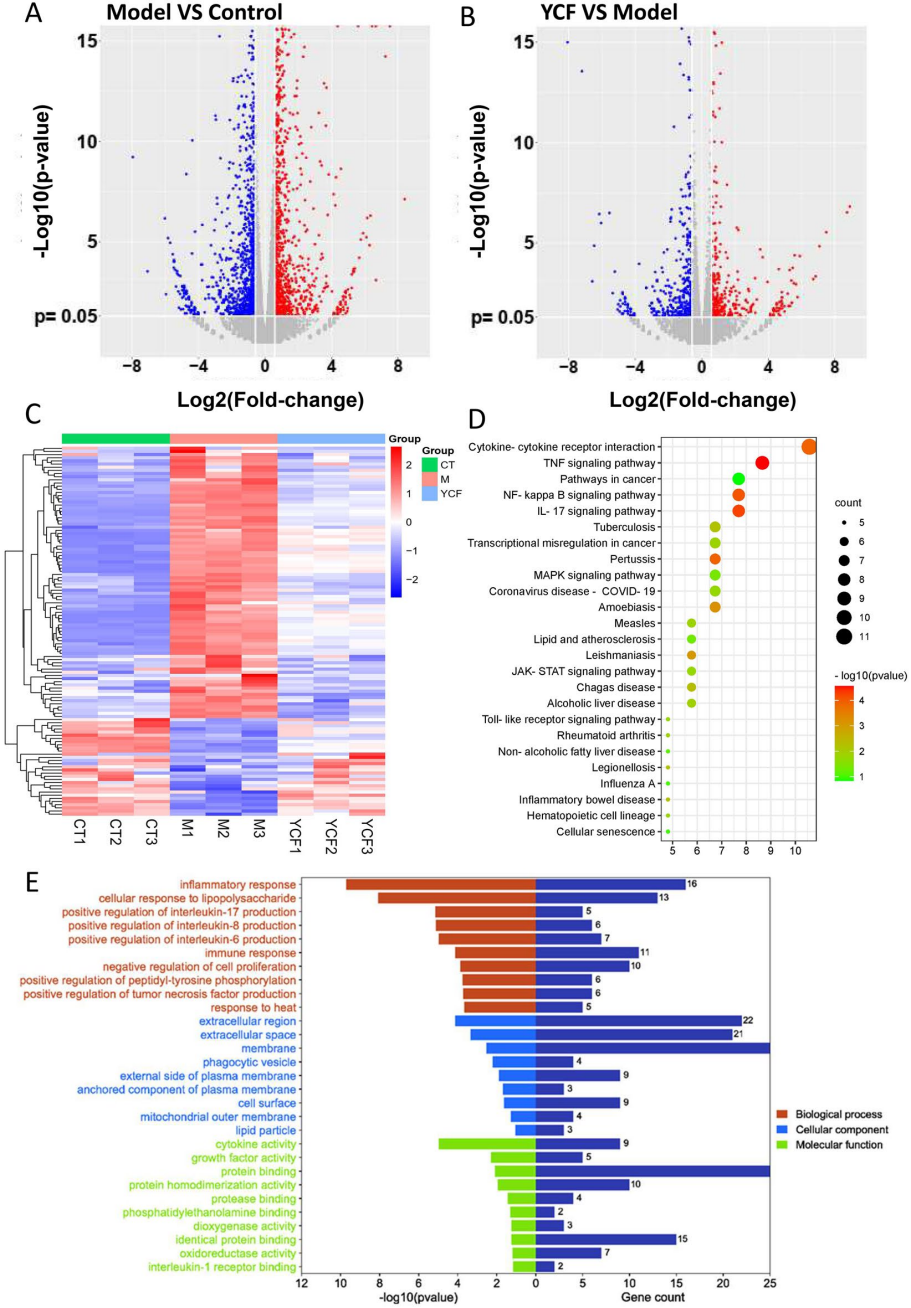
The analysis of reversal genes in M1 macrophages. **A** Volcano map of differentially expressed genes between the Model group and the Control group. **B** Volcano map of differentially expressed genes between the YCF group and the Model group. **C** Heatmap of the reversal genes from the differentially expressed genes. **D** Kyoto Encyclopedia of Genes and Genomes (KEGG) analysis of the reversal genes. **E** Biological process analysis, Cellular component analysis, and Molecular function analysis of the reversal genes

To detect gene expression changes related to M1 polarization that were regulated by YCF5 in macrophages, we used RNA-seq to examine the differentially expressed genes in YCF5-treated M1 macrophages. Compared with the normal macrophages, 1289 differentially expressed genes, including 752 up-regulated genes and 537 down-regulated genes, were identified in macrophages in response to LPS- and IFN-γ treatment; Compared to the LPS- and IFN-γ-induced macrophages, 315 differentially expressed genes, including 157 up-regulated genes and 158 down-regulated genes, were identified in YCF5-treated macrophages (Fig. 4A). YCF treatment reversed 117 changes in 1289 genes (Fig. 4B). The DAVID database was used to perform KEGG and GO analyses on the 117 possible target genes. The top 25 KEGG pathways included Cytokine-cytokine receptor interaction, IL-17 signaling pathway, Toll-like receptor signaling pathway, TNF signaling pathway, MAPK signaling pathway, mTOR signaling pathway, NF-κB signaling pathway, JAK-STAT signaling pathway, and Pathways in cancer (Fig. 4C). Furthermore, 141 GO terms were identified, and the top 10 terms were mainly associated with immune and inflammatory responses, regulating the production of various interleukins and tumor necrosis factors (Fig. 4D). These results indicated that the reversal genes regulated by YCF5 are mainly associated with inflammation.

### Integrated analysis of network pharmacology and transcriptomic data

**Fig. 5:**
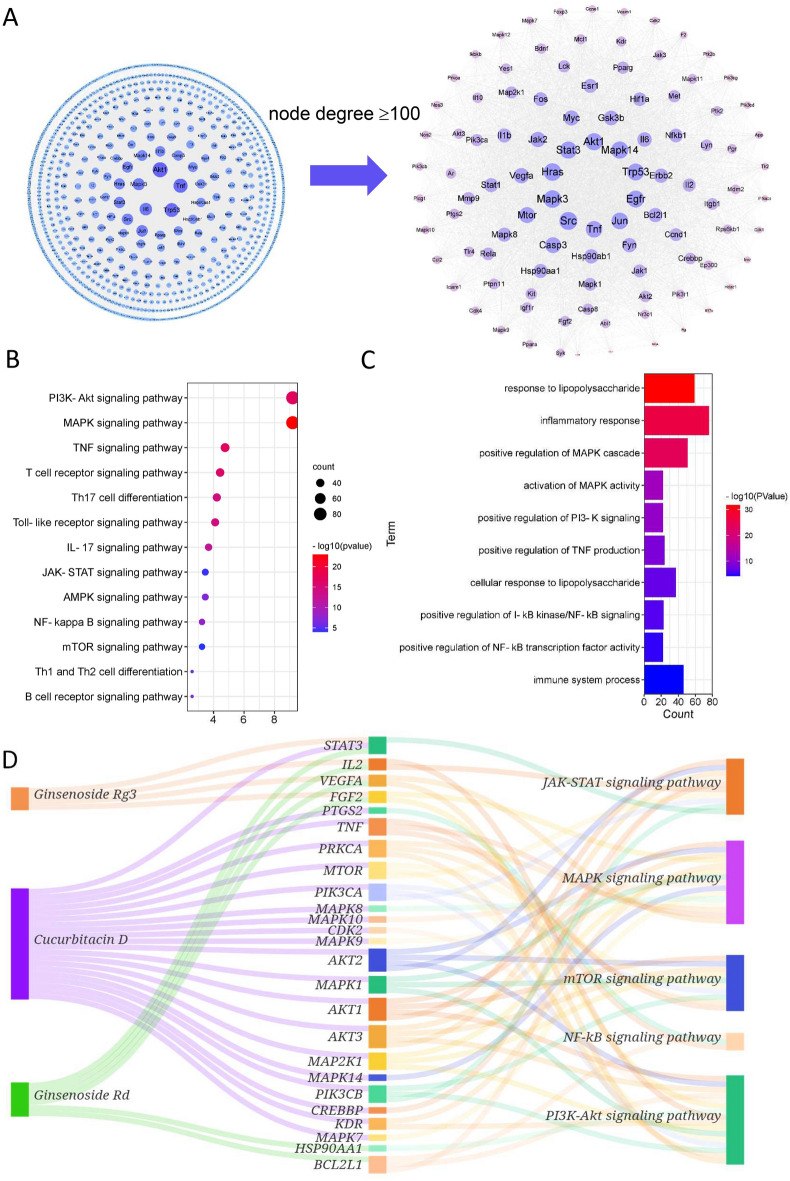
Integrative analysis of network pharmacology and transcriptomic data. **A** Protein–protein interaction network of the targets of YCF5 active components and the reversal genes. **B** Kyoto Encyclopedia of Genes and Genomes (KEGG) pathways closely associated with inflammation for all nodes. **C** Biological process analysis of gene ontology for the targets of YCF5. **D** Sankey diagram of the “YCF active compound-target-pathway” and the related targets of three pathways that are closely associated with the inflammatory response

Based on the results described above, YCF5 is an effective fraction of YCF that exerts anti-inflammatory effects. To further explore the molecular mechanisms of YCF5 on M1 polarization, a PPI network of YCF5-related targets and reversal genes was constructed. This network consisted of 1016 nodes and 22,219 edges (Fig. 5A). The KEGG and GO enrichment analysis of these nodes is showed in Fig. 5B; 194 KEGG pathways, including TNF signaling pathway, T cell receptor signaling pathway, Th1 and Th2 cell differentiation, Toll-like receptor signaling pathway, Th17 cell differentiation, IL-17 signaling pathway, B cell receptor signaling pathway, JAK-STAT signaling pathway, AMPK signaling pathway, HIF signaling pathway, mTOR signaling pathway, MAPK signaling pathway, PI3K-Akt signaling pathway, and NF-κB signaling pathway, may be the important mechanisms by which YCF5 promotes M1 macrophage polarization. Moreover, 1628 enriched biological processes were predominantly associated with inflammatory response (Fig. 5C). Therefore, YCF5, the effective fraction of YCF, exerts anti-inflammatory effects by inhibiting M1 polarization through the pathways listed above (see Table 4).

**Table 4:** Settings for protein parameters

Protein	Center_x	Center_y	Center_z	Size_x	Size_y	Size_z
AKT1	− 3.319	2.217	− 9.571	90	90	90
BCL2L1	4.099	− 0.36	14.021	80	80	80
JUN	8.608	4.411	− 11.651	126	126	126
mTOR	25.261	18.58	− 22.942	126	126	126
STAT3	108.215	69.055	24.533	60	60	60
VEGFA	− 16.811	− 12.938	− 6.955	60	60	60
JAK1	186.964	194.713	159.771	40	40	40

### Molecular docking analysis of YCF5 compounds and their targets proteins

**Fig. 6:**
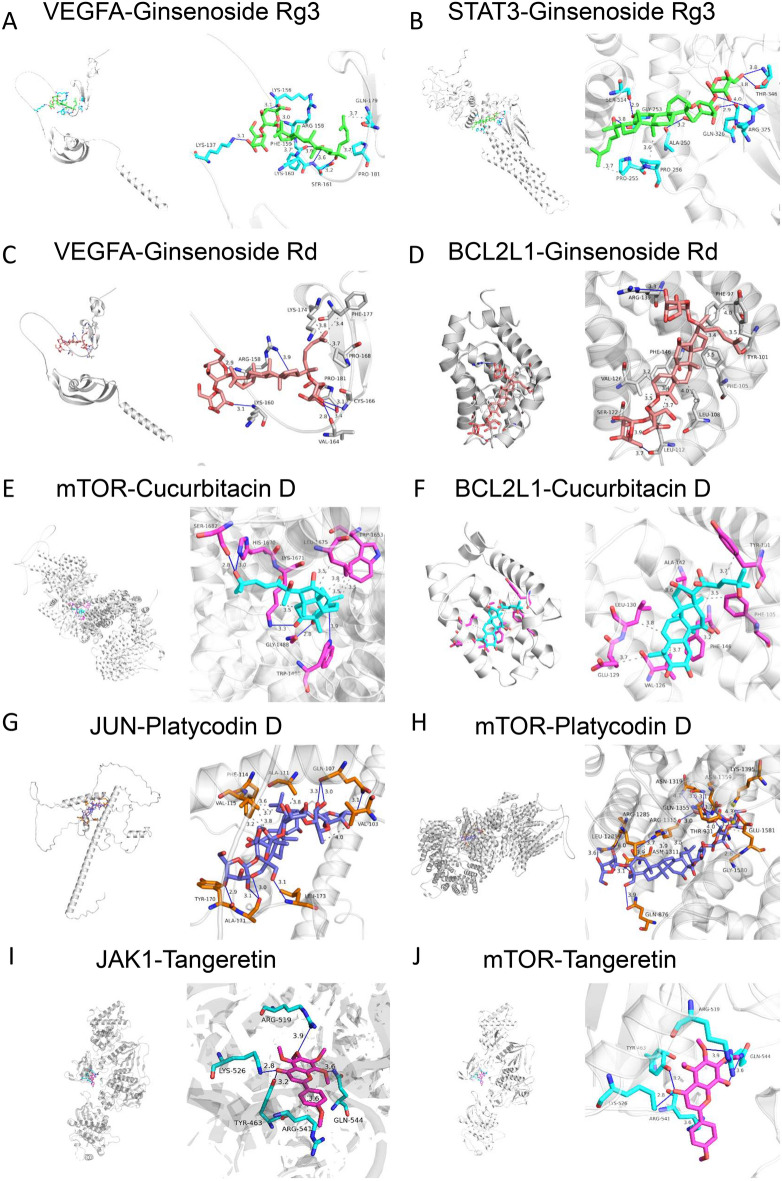
Molecular docking analysis. Molecular docking of VEGFA, STAT3, BCL2L1, mTOR, JUN, JAK1, and AKT1 with 20(s)-Ginsenoside Rg3, Ginsenoside Rd, Cucurbitacin D, Platycodin D, and Tangeretin. These amino acids are all close to the binding sites for the JAK-STAT, mTOR, MAPK, PI3K-Akt, and NF-κB signaling pathways

Multiple signaling pathways, including the NF-κB, mTOR, PI3K/AKT, MAPK, and JAK-STAT signaling pathway, were closely related to the inflammatory response in an integrated analysis of network pharmacology and transcriptomics data. Moreover, 189 enriched targets in these pathways were associated with 81 active components of YCF5, such as Polygalacin D, Platycodin D, Platycoside K, Cucurbitacin D, Astersaponin A, Ginsenoside Rg5, Ginsenoside Rg3, and Ginsenoside Rd. We selected three components and their related targets and pathways to establish a component-target-pathway Sankey diagram (Fig. 5D). To determine if the major components of YCF played a substantial role in the anti-inflammatory pathways, molecular docking was also performed. The primary targets were selected from the JAK-STAT, mTOR, MAPK, PI3K-Akt, and NF-κB signaling pathways, including VEGFA, STAT3, BCL2L1, mTOR, JUN, JAK1, and AKT1, for further molecular docking. The key active components of YCF5 had excellent affinities for the proteins in the anti-inflammatory pathways (Fig. 6 and Table 5).

**Table 5:** Binding energy of molecular docking among compounds and proteins

Proteins	Components	Binding energy
VEGFA	20(s)-Ginsenoside Rg3	− 7 kcal/mol
STAT3	20(s)-Ginsenoside Rg3	− 7.3 kcal/mol
BCL2L1	Ginsenoside Rd	− 7 kcal/mol
VEGFA	Ginsenoside Rd	− 7.1 kcal/mol
BCL2L1	Cucurbitacin D	− 7.8 kcal/mol
mTOR	Cucurbitacin D	− 8.5 kcal/mol
JUN	Platycodin D	− 5.4 kcal/mol
mTOR	Platycodin D	− 10.9 kcal/mol
JAK1	Tangeretin	− 6.3 kcal/mol
AKT1	Tangeretin	− 7.4 kcal/mol

### Experimental validation of YCF active compounds and related-pathways

**Fig. 7:**
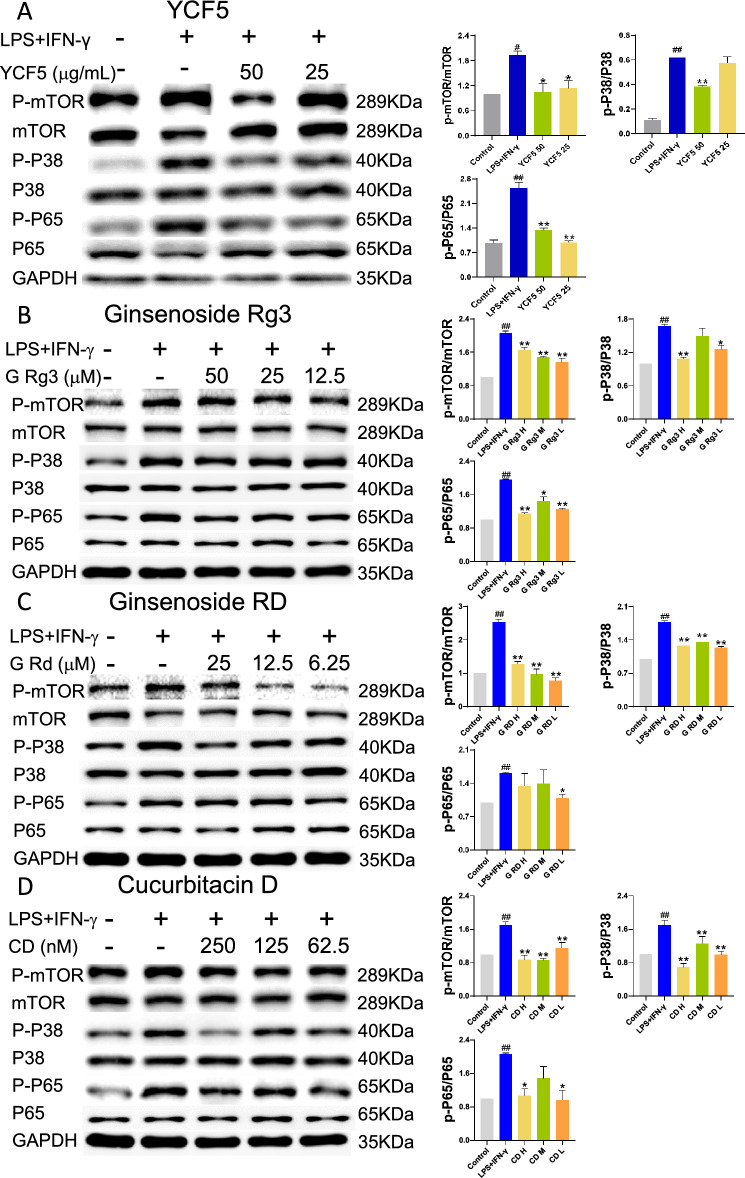
Experimental validation of YCF active compounds and related pathways. **A** Protein expression levels (measured by western blot) of p-P38, P38, p-P65, P65, p-mTOR, mTOR, and GAPDH in M1 macrophages after treatment with different concentrations of YCF5. **B** Protein expression levels of p-P38, P38, p-P65, P65, p-mTOR, mTOR, and GAPDH in M1 macrophages after treatment with different concentrations of Ginsenoside Rg3. **C** Protein expression levels of p-P38, P38, p-P65, P65, p-mTOR, mTOR, and GAPDH in M1 macrophages after treatment with different concentrations of Ginsenoside RD. **D** Protein expression levels of p-P38, P38, p-P65, P65, p-mTOR, mTOR, and GAPDH in M1 macrophages after treatment with different concentrations of Cucurbitacin D. All data are presented as the mean ± SD (*n* = 3). ^#^*p* < 0.05, ^##^*p* < 0.01 *vs*. Control group; ^*^p < 0.05, ^**^p < 0.01 *vs*. Model group

To validate the results, we evaluated the effects of YCF5 and the three compounds on the identified pathways. MAPK and NF-κB are thought to play key regulatory roles in regulating the expression of pro-inflammatory factors [22]. Activation of mammalian target of rapamycin complex 1 (mTORC1) is also regulated by the inflammatory response [23]. Our results indicated that YCF5 significantly suppressed the phosphorylation of mTORC1, MAPK p38 and NF-κB p65. Moreover, the active compounds of YCF5, including Ginsenoside Rg3, Ginsenoside Rd, Cucurbitacin D, also significantly reduced the phosphorylation level of mTORC1, MAPK p38 and NF-κB p65 in M1 macrophages (Fig. 7). Thus, YCF5 contains various active compounds that can inhibit LPS- and IFN-γ-induced M1 polarization by suppressing these pathways.

## Discussion

Silicosis, caused by prolonged inhalation of crystalline silica, is an incurable progressive fibrotic pulmonary disease with chronic inflammation [24]. Due to the recent rise of artificial stone (a material containing more than 90% crystalline silica) in the global building materials industry, silicosis, which has high morbidity and mortality rates in developing countries, is quietly increasing worldwide [25]. However, no specific drug is available to treat silicosis, and lung transplantation is the only effective treatment. Thus, novel drugs are urgently needed to suppress the progression of silicosis. YCF is a Chinese medicine for pneumoconiosis patients with yin deficiency and dryness heat syndrome or dryness invading lung syndrome. Previous studies demonstrated that YCF effectively improves lung function and alleviates clinical symptoms, such as coughing, shortness of breath, and dyspnea. The present study suggests that YCF significantly improves lung tissue damage and inhibits inflammation and fibrosis in rats with silicosis. Moreover, YCF can significantly inhibit macrophage M1 polarization in vivo and in vitro. By integrating transcriptomic analysis and network pharmacology, we found that YCF has a variety of active components, including ginsenoside Rg3, platycoside D, cucurbitacin D, which can bind to multiple targets and regulate various signaling pathways, including the mTOR, PI3K/AKT, JAK/STAT, MAPK, and NF-κB signaling pathway. These pathways may be involved in the mechanisms by which YCF inhibits inflammation and suppresses the progression of silicosis.

Although the pathogenesis of silicosis remains unclear, increasing evidence suggests that silica-induces persistent pulmonary inflammation, causing tissue damage and fibrosis [26, 27]. In response to silica exposure, activated macrophages polarize into M1 macrophages, which release large amounts of pro-inflammatory mediators, such as IL-1β, IL-6, and TNF-α, that further trigger the inflammatory cascade, leading to tissue damage and fibrosis [28–30]. Thus, inhibition of the macrophage-induced inflammatory response may effectively ameliorate silicosis. A previous study demonstrated that bone marrow mesenchymal stem cells exert therapeutic effect in rats with silicosis by ameliorating inflammation and reducing the release of inflammatory cytokines [31]. Moreover, dioscin protects against crystalline silica-induced lung inflammation by suppressing the production of inflammatory factors and inhibiting the activation of macrophages [32]. In our study, YCF treatment significantly inhibited pulmonary inflammation in silica-exposed rats, by reducing inflammatory cell infiltration, and decreasing the secretion of inflammatory chemokines, including TNF-α, IL-1β, and IL-6. YCF also exhibited remarkable anti-fibrotic effects by inhibiting collagen deposition. Moreover, YCF decreased the amount of M1 macrophages in the lung tissue of rats with silicosis. Therefore, we speculate that YCF may exert anti-inflammatory properties via inhibiting M1 macrophage polarization.

To clarify the therapeutic mechanisms of YCF, we separated YCF into five fractions using macroporous resins, and examined the anti-inflammatory effects of these fractions on LPS and IFN-γ-induced M1 macrophages. YCF5 substantially inhibited the production of pro-inflammatory cytokines. These results indicate that YCF5 may be the essential fraction that exerts anti-inflammatory effects on M1 macrophages to attenuate inflammation and delay the progression of silicosis. However, the underlying mechanism of YCF5 attenuation of inflammation via altered macrophage polarization needs to be explored. Thus, we obtained the main active components of YCF5 by MS, network pharmacology and transcriptomics revealed the potential anti-inflammatory mechanisms of YCF5.

Traditional Chinese formulas exert curative effects through multiple compounds and targets with synergistic effects. The holistic and systematic characteristics of network pharmacology are consistent with the “holism concept” in traditional Chinese medicine. Network pharmacology can be used to construct a “compound-target-pathway” network to understand the overall perspective of effective substances and their mechanisms. In recent years, the combination of transcriptomics and network pharmacology has proven to be an effective approach for exploring the therapeutic mechanisms of traditional Chinese prescriptions. Using network pharmacology analysis, we identified the core targets of YCF5, including AKT1, JAK2, MAPK3, STAT3, MYC, EGFR, mTOR, VEGFR, and TNF. These signaling pathways and biological processes, including Toll-like receptor, TNF, and IL-17 signaling pathways, are strongly linked to immunological responses and inflammation.

AKT1, a serine/threonine-protein kinase, is activated through the PI3-kinase pathway to regulate cellular survival signals in response to growth factors and cytokines [33]. The activation of AKT1 accelerates the degradation of IκB and leads to the phosphorylation of NF-κB p65, which promotes the transcriptional activity of NF-κB [34]. Mitogen-activated protein kinase 3 (MAPK3 or ERK) is involved in cell proliferation, growth, migration, metabolism, and transcription [35]. MAPK3 (ERK1) levels are dramatically elevated in the peripheral blood mononuclear cells of patients with silicosis, and crystalline silica may accelerate the release of ROS [36]. ROS further activate the inflammasome through MAPK3 and phosphorylate Ser276 of p65 NF-κB and Ser641 and Ser643 of HIF-1α, to promote the development of silicosis [36–38]. JAK2 and STAT3, crucial proteins in the JAK/STAT signaling pathway, play a significant role in regulating macrophage polarization in silicosis; inhibiting the expression of these proteins can delay the progression of silicosis [39]. PI3K-Akt targets mTOR, a crucial component of the rapamycin (mTOR) signaling pathway [40, 41]. Furthermore, mTOR increases autophagy and aggravates the progression of pulmonary fibrosis in silicosis under the regulation of AMP-activated protein kinase (AMPK) [42]. VEGFA, a ligand for the VEGF receptor, plays an important role in angiogenesis and inflammation [43]. Manipulating VEGFA inhibits fibrosis factor release, suppressing the expression of TGF-β and α-SMA in the lung tissue of rats with silicosis [44]. Thus, the targets of YCF5 play vital roles in the inflammatory response, and modulating these targets can delay the progression of silicosis.

Using transcriptomic analysis, we identified 117 reversal genes for YCF5 inhibition of M1 macrophage polarization. These reversal genes are primarily involved in immunological and inflammatory responses, including the regulation of interleukins and tumor necrosis factor production. By integrating network pharmacology and transcriptomic analysis, we constructed a PPI network of YCF5 targets and reversal genes. The anti-inflammatory mechanisms of YCF5 were associated with various targets, including AKT1, TNF, TRP53, IL6, MAPK3, and JUN, and signaling pathways, including the including PI3K-Akt, MAPK, TNF, JAK-STAT, mTOR, NF-κB, and AMPK signaling pathways. These signaling pathways play critical roles in regulating inflammation. For example, MAPKs and NF-κB regulate the expression of pro-inflammatory factors [22]. The activation of mTORC1 also influences the inflammatory response [23]. The targets enriched in these pathways are related to 81 active components, which may be the main active components of YCF. In vitro, we confirmed the activity of YCF5 and the active components of YCF, including ginsenoside Rg3, Ginsenoside Rd, and Cucurbitacin D, which suppressed the activation of these signaling pathways.

## Conclusions

Our study demonstrated that YCF treatment improved the pathological changes, inflammatory response, and fibrosis in rats with silicosis, probably by inhibiting M1 macrophage polarization. Moreover, network pharmacology, transcriptomics, molecular docking, and in vitro experiments showed that YCF contains multiple effective compounds with various targets that exert anti-inflammatory effects by inhibiting the pathway networks, such as mTOR, MAPK, and NF-κB signaling pathways. Although this study provides an explanation for the anti-silicosis effects of YCF, there are several limitations to this study. For instance, 101 active compounds identified in YCF5 exert anti-inflammatory effects; however, the identity of the specific active substance of YCF with beneficial effects on silicosis is not known. In future work, we will perform the effective-constituent compatibility-based analysis to identify the critical ingredients from YCF and then form the effective-constituent compatibility (ECC) of JCF, which has the potential bioactive equivalent of JCF [45]. Moreover, we will explore the anti-silicosis mechanisms and potential targets of the ECC of YCF.

## Data Availability

The datasets used or analysed during the current study are available from the corresponding author on reasonable request.
